# VT-MFLV: Vision–Text Multimodal Feature Learning V Network for Medical Image Segmentation

**DOI:** 10.3390/jimaging11120425

**Published:** 2025-11-28

**Authors:** Wenju Wang, Jiaqi Li, Zinuo Ye, Yuyang Cai, Zhen Wang, Renwei Zhang

**Affiliations:** College of Publishing, University of Shanghai for Science and Technology, Shanghai 200093, China

**Keywords:** medical image segmentation, vision-text, multimodal feature learning

## Abstract

Currently, existing multimodal segmentation methods face limitations in effectively leveraging medical text to guide visual feature learning. They often suffer from insufficient multimodal fusion and inadequate accuracy in fine-grained lesion segmentation accuracy. To address these challenges, the Vision–Text Multimodal Feature Learning V Network (VT-MFLV) is proposed. This model exploits the complementarity between medical images and text to enhance multimodal fusion, which consequently improves critical lesion recognition accuracy. VT-MFLV introduces three key modules: Diagnostic Image–Text Residual Multi-Head Semantic Encoding (DIT-RMHSE) module that preserves critical semantic cues while reducing preprocessing complexity; Fine-Grained Multimodal Fusion Local Attention Encoding (FG-MFLA) module that strengthens local cross-modal interaction; and Adaptive Global Feature Compression and Focusing (AGCF) module that emphasizes clinically relevant lesion regions. Experiments are conducted on two publicly available pulmonary infection datasets. On the MosMedData dataset, VT-MFLV achieved Dice and mIoU scores of 75.61 ± 0.32% and 63.98 ± 0.29%. On the QaTa-COV1 dataset, VT-MFLV achieved Dice and mIoU scores of 83.34 ± 0.36% and 72.09 ± 0.30%, both reaching world-leading levels.

## 1. Introduction

Medical image segmentation is a medical image processing technique [1], whose primary objective is to analyze and process medical images through computational algorithms in order to precisely delineate lung tissues and effectively distinguish lesion regions (including pulmonary nodules [2], tumors [3], and inflammations [4]) from normal tissues. The segmentation results offer clinicians accurate lesion localization and quantitative assessment. These capabilities collectively improve diagnostic efficiency and treatment precision. Moreover, they reduce the risk of misdiagnosis, enhance therapeutic decision-making, and facilitate visual monitoring of disease progression. Consequently, lung segmentation methods hold substantial research significance and practical value.

Due to the high complexity of pulmonary infection regions, accurate segmentation in current practice is generally achieved through manual or semi-manual approaches. However, deep learning techniques enable automated and rapid processing of large-scale medical images, which not only enhances diagnostic efficiency but also alleviates the workload of radiologists. Therefore, medical image segmentation based on deep learning has become a prominent research focus.

According to the type and source of input data, deep learning-based medical image segmentation algorithms can be categorized into unimodal and multimodal methods.

### 1.1. Unimodal Medical Image Segmentation Methods

Unimodal medical image segmentation methods utilize only medical images (such as CT or X-ray) to automatically identify regions of pulmonary lesions through feature extraction and segmentation algorithms, thereby supporting accurate diagnosis and treatment. Based on the sample size and the number of datasets, unimodal segmentation approaches can be categorized into three types: small-sample dataset methods, large-sample single-dataset methods, and large-sample multi-dataset methods.

(1) Small-sample dataset methods

Small-sample methods are commonly built on U-Net variants enhanced with attention modules, dense connections, or multi-scale feature encoders [5,6,7,8,9,10]. These techniques aim to highlight small lesions and improve feature utilization when only limited annotations are available. However, these models are usually trained on only a few hundred images, which restricts feature diversity and leads to overfitting. Their performance drops when facing subtle lesion structures, blurred boundaries, or diverse clinical environments. Thus, despite targeted improvements, generalization remains limited under small-sample conditions.

(2) Large-Sample Single-Dataset Segmentation Methods

Large-sample single-dataset methods expand model capacity using deeper backbones, transformer units, hybrid loss designs, or pretrained encoders [11,12,13,14,15,16,17,18,19,20,21,22,23,24,25,26,27,28]. They achieve strong results on widely used datasets such as LUNA16, LIDC-IDRI, and TCGA. Yet each model relies on a single data source, often from one imaging system or disease type. As a result, their performance decreases when transferred across devices, institutions, or disease distributions. Many of these approaches also increase computational cost and inference latency, which limits clinical deployment. Therefore, single-dataset training still struggles with broad generalization in real practice.

(3) Large-sample multi-dataset segmentation methods

Multi-dataset approaches employ domain adaptation, adversarial learning, and multi-scale fusion to reduce domain shift and improve robustness [29,30,31,32,33,34,35,36,37,38]. They are trained on diverse imaging conditions and demonstrate better cross-center stability. However, these improvements often require complex training schedules and significant computational resources. Sensitivity to image contrast, noise, and acquisition variations may persist. Most importantly, these models still rely solely on visual information, which makes it difficult to capture diagnostic intent and resolve ambiguous lesion boundaries. This motivates incorporating multimodal knowledge to enhance lesion understanding and clinical interpretability.

Although unimodal approaches have demonstrated promising performance, their dependence on single-source information inherently restricts feature diversity. As a result, these methods often struggle to adapt to lesion variability, exhibit reduced robustness under complex imaging conditions, and show limited generalization across heterogeneous clinical datasets. In contrast, multimodal methods benefit from complementary information derived from visual and textual representations, enabling more comprehensive feature modeling and consequently improving segmentation reliability in real-world scenarios.

### 1.2. Multimodal Medical Image Segmentation Methods

Compared with unimodal segmentation methods, multimodal medical image segmentation approaches integrate multiple data types, enabling feature extraction and model training from heterogeneous modalities. This integration facilitates more accurate segmentation and identification of pulmonary lesion regions.

Multimodal approaches can generally be divided into two categories. The first combines different types of imaging data, while the second integrates additional modalities (e.g., textual radiological descriptions) with imaging data for segmentation. CM-SegNet (Convolution and Multilayer Perceptron-based automatic segmentation approach for medical images) [39] enhances segmentation accuracy and improves lesion detail recognition by exploiting the complementarity of different medical imaging modalities, such as CT, MRI, and mp-MRI. However, discrepancies in resolution, perspective, and alignment among modalities can limit model performance. MTSSL (Multi-Task Semi-Supervised Learning) [40] fuses CT images, structured labels, and anatomical priors to improve model robustness. Nevertheless, anatomical priors cannot provide the flexible, dynamic semantic information offered by textual descriptions, which are better suited to capture diverse lesion characteristics. In addition, anatomical priors often rely on expert annotations or expert systems, requiring extra time and resources. In contrast, LViT (Language meets Vision Transformer) [41] integrates textual descriptions with medical images, where information about lesion location, size, and quantity augments visual features with richer contextual knowledge. This modality is particularly advantageous for clinical practice in detecting subtle or inconspicuous lesions. Moreover, textual medical records are usually generated alongside patients’ imaging data, incurring no additional cost.

Compared with unimodal medical image segmentation methods, existing multimodal approaches provide more comprehensive information by combining heterogeneous modalities, thereby overcoming the limitations of single-source data in lesion recognition. However, current multimodal approaches often fail to fully exploit the guiding role of textual descriptions in visual feature learning, and they suffer from insufficient feature fusion as well as inadequate precision in fine-grained lesion region identification. To address these limitations, this thesis proposes the Vision–Text Multimodal Feature Learning V Network for Medical Image Segmentation (VT-MFLV). The contributions of this work are summarized as follows:

1. VT-MFLV, a vision–text multimodal feature learning V-shaped network for medical image segmentation, introduces three core components: diagnostic image–text sequence multi-head residual semantic encoding (DIT-RMHSE), multimodal fusion local attention fine-grained feature encoding (FG-MFLA), and multimodal global feature adaptive compression focusing (AGCF). Experimental results demonstrate that VT-MFLV achieves state-of-the-art segmentation performance on both the MosMedData+ and QaTa-COV19 datasets.
2. The Diagnostic Image–Text Sequence Multi-Head Residual Semantic Encoding Module (DIT-RMHSE) transforms medical text into high-dimensional semantic representations and captures rich contextual information. It eliminates the need for token type embeddings while effectively modeling complex semantic relationships, thereby improving lesion localization and enhancing multimodal fusion flexibility.
3. The multimodal fusion local attention fine-grained feature encoding module (FG-MFLA) combines a multi-head attention mechanism with local attention masks and introduces a cross-modal fusion unit (CMFU). This module optimizes cross-modal feature integration and enhances representation learning. Consequently, it effectively addresses the issue of inaccurate local detail recognition caused by insufficient multimodal fusion.
4. The multimodal global feature adaptive compression focusing module (AGCF) employs a squeeze-and-excitation refinement strategy (SER) and pixel-level feature enhancement to adaptively adjust channel weights. By suppressing redundant background information and focusing on critical regions, AGCF alleviates challenges commonly encountered in medical image segmentation, such as blurred boundaries and small lesion volumes. This significantly improves segmentation accuracy and stability.

## 2. Methodology

**Figure 1:**
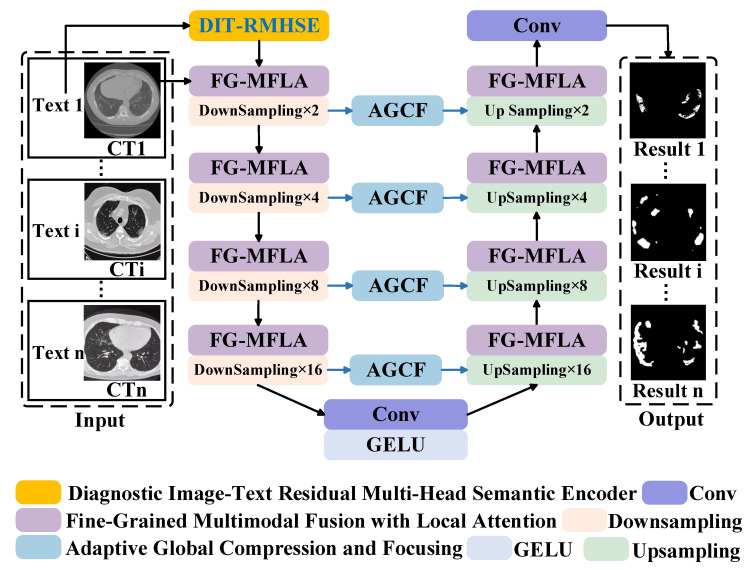
Vision–Text Multimodal Feature Learning V Network Structure.

The proposed Vision–Text Multimodal Feature Learning V Network for Medical Image Segmentation (VT-MFLV) takes medical images and their corresponding textual descriptions (including lesion location, number, and extent of infection) as input, thereby generating accurate segmentation results of lesion regions. The overall framework is illustrated in Figure 1. VT-MFLV primarily consists of three components: Diagnostic Image–Text Residual Multi-Head Semantic Encoding (DIT-RMHSE), Fine-Grained Multimodal Fusion Local Attention (FG-MFLA), and Adaptive Global Feature Compression and Focusing (AGCF). DIT-RMHSE leverages a strong contextual modeling capability to transform medical text into high-dimensional semantic representations. Without relying on Token Type Embedding, it effectively captures complex semantic relationships, enhances lesion localization ability, and improves the flexibility of multimodal fusion (Section 2.1). FG-MFLA integrates multi-head attention with a local feature enhancement strategy, fusing image and text features while extracting multi-scale information from images. This process effectively optimizes cross-modal feature fusion and strengthens spatial information modeling (Section 2.2). AGCF employs a channel compression excitation refinement strategy (SER) combined with a pixel-level feature enhancement strategy to adaptively adjust feature channel weights, thereby improving the model’s sensitivity to key regions (Section 2.3). Text features processed by DIT-RMHSE and medical image features are progressively compressed into multi-scale fusion representations through four layers of FG-MFLA and down-sampling modules. The compressed features obtained after the fourth down-sampling are further refined via convolution and activation functions. Together with multi-scale global-local key feature representations extracted by AGCF, these features are fed into four symmetric up-sampling, FG-MFLA, and convolution operations, ultimately producing accurate lesion region segmentation maps. In the Appendix A, we provide representative examples of unified textual annotations for the mathematical symbols and variables used in our method.

### 2.1. Diagnostic Image–Text Residual Multi-Head Semantic Encoder

**Figure 2:**
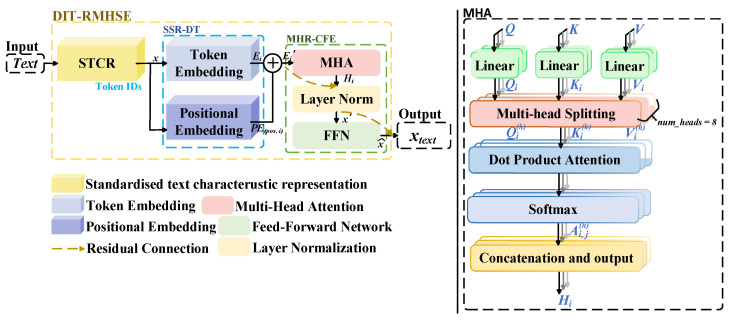
Diagnostic Image–Text Residual Multi-Head Semantic Encoder.

Text associated with diagnostic medical images provides richer contextual information for medical image segmentation. When dealing with implicit or hardly noticeable lesions, textual descriptions of lesion location, size, and type effectively complement image information. However, existing feature processing methods for medical record text [41] exhibit weak capabilities in local feature extraction and involve high computational costs, thereby reducing efficiency and practicality. To address these issues, this thesis proposes the Diagnostic Image–Text Residual Multi-Head Semantic Encoder (DIT-RMHSE). Without relying on Token Type Embedding based on the experience of RoBERTa [42], this method transforms diagnostic text generated during clinical practice into high-dimensional semantic representations and captures its abundant contextual information. DIT-RMHSE consists of three main components: Standardized Text Characteristic Representation (STCR), Sequential Semantic Representation of Diagnostic Text (SSR-DT), and Multi-Head Residual Context Fusion Encoding (MHR-CFE), as illustrated in Figure 2.

#### 2.1.1. Standardized Text Characteristic Representation

To convert raw text into high-dimensional vector representations interpretable by the model, processes such as tokenisation, index mapping, and special symbol extension must be performed. This procedure is referred to as Standardized Text Characteristic Representation (STCR). Specifically, based on a predefined vocabulary, the raw text sequence $Text=\left\{c_{1},c_{2},\ldots ,c_{I},\ldots {,c}_{L}\right\}$ is segmented into subword units, resulting in a tokenised sequence $Text=\left\{t_{1},t_{2},\ldots ,t_{i},\ldots ,t_{N}\right\}$. Here, $c_{i}$ denotes the i-th character, L is the total number of original characters, t_i denotes the i-th token, and N represents the number of tokens after tokenisation. Each token is then mapped to its index in the vocabulary, forming an index sequence, with special symbols [CLS] and [SEP] added at the beginning and the end. Thus, the standardized text representation is expressed as $ID=\{ID\left(\left[CLS\right]\right),ID\left(t_{1}\right),\ldots ,ID\left(t_{i}\right),\ldots ,ID\left(t_{N}\right),ID\left(\left[SEP\right]\right)\}$, where ID (tᵢ) denotes the unique index position of the i-th token in the vocabulary. This index sequence is subsequently converted into one-hot vectors $x=\left[x_{1},\ldots ,x_{i},\ldots ,x_{N}\right],where\;x_{i}\in R^{V}$, $V\;denotes\;the\;vocabulary\;size,\;and\;x_{i}$ corresponds to the one-hot vector of the i-th token. The one-hot representation is then used as the input to the embedding layer for subsequent deep encoder modeling and semantic extraction.

#### 2.1.2. Sequential Semantic Representation of Diagnostic Text

This process consists of three components: token embeddings, positional embeddings, and embedding fusion.

1. Token Embeddings

The input one-hot vector $x_{i}$ is mapped into a dense word vector representation $E_{i}$ through the embedding matrix $W_{T}\in R^{d\times V}$, where d denotes the dimension of the word embedding vector and V represents the vocabulary size. The mapping process is shown in Equation (1):

(1) $$E_{i}=W_{T}x_{i}$$

2. Position Embeddings

The word vector representation $E_{i}$ obtained solely from token embeddings cannot provide positional information. Therefore, positional embeddings are introduced to explicitly model the sequential order among positions in the text. Positional encoding is computed based on the index position pos of the current feature $x_{i}$ in the sequence and the index i of the embedding dimension, denoted as ${PE}_{\left(pos,i\right)}$. Specifically, when the embedding dimension index i is even, the positional encoding value is generated using a sine function, while for odd i, it is generated using a cosine function, as shown in Equation (2). The sine function facilitates capturing local variation patterns, whereas the cosine function, with its orthogonality property, enhances feature discriminability. Together, they construct position-dependent representations with periodic variation, enabling the model to perceive semantic differences across positions.

(2) $${PE}_{\left(pos,i\right)}=\left\{\begin{array}{c} \sin\left(\frac{pos}{10000^{\frac{2i}{d}}}\right)\;,\;if\;i\;is\;even\\ \cos\left(\frac{pos}{10000^{\frac{2i}{d}}}\right),\;\mathrm{if}\;i\;\mathrm{is}\;\mathrm{odd} \end{array}\right.$$

In this equation, d denotes the total dimension of the embedding features, and the denominator term $10000^{\frac{2i}{d}}$ adjusts the encoding frequency across different dimensions. This design enables lower dimensions to capture local dependencies, while higher dimensions model long-range relationships, thereby significantly enhancing the model’s capacity for global context representation.

3. Embedding Fusion

The input $E_{i}^{\prime }$ of the Sequential Semantic Representation of Diagnostic Text (SSR-DT) is obtained by performing element-wise addition between the output $E_{i}$ of the token embeddings and the output ${PE}_{\left(pos,i\right)}$ of the positional embeddings, as shown in Equation (3). This operation allows the embedding vector of each token $x_{i}$ to be fused with its corresponding positional information. Consequently, the semantic features of the vocabulary are preserved while simultaneously incorporating positional dependencies within the context.

(3) $$E_{i}^{\prime }=E_{i}+{PE}_{\left(pos,i\right)}$$

#### 2.1.3. Multi-Head Residual Context Fusion Encoding (MHR-CFE)

In the process of text feature processing, traditional methods often struggle to comprehensively capture long-range dependencies. To address this limitation, Multi-head Residual Context Fusion Encoding (MHR-CFE) is introduced. MHR-CFE consists of three main components: multi-head attention, residual normalization, and feedforward residual fusion.

1. Multi-Head Attention

Multi-head attention enhances contextual awareness by computing the relationships between each token and all other tokens in parallel, thereby improving the model’s ability to handle complex linguistic structures and long-range dependencies. Its implementation comprises four steps: linear transformation, attention weight calculation, weighted summation, and parallel attention integration.

(1) Linear Transformation

In the encoder layer of the Diagnostic Image–Text Residual Multi-Head Semantic Encoder (DIT-RMHSE), the embedding vector $E_{i}^{\prime }$ at position i has a relatively high dimensionality. Directly processing such vectors would lead to computational redundancy and inefficiency. Therefore, $E_{i}^{\prime }$ is first projected into three subspaces—query (Q), key (K), and value (V)—through linear transformations, yielding the corresponding representations $Q_{i}$, $K_{i}$, and $V_{i}$, as shown in Equation (4). The vectors of all positions iii are then aggregated to form the matrices Q, K, and V, expressed as: $Q={\left[\;Q_{1},...,Q_{i},...,Q_{\tilde{N}}\right]}^{T}$, $K={\left[\;K_{1},...,K_{i},...,K_{\tilde{N}}\right]}^{T}$, $V={\left[\;V_{1},...,V_{i},...,V_{\tilde{N}}\right]}^{T}$.

(4) $$\left\{\begin{array}{c} Q_{i}=E_{i}^{\prime }W_{Q}\;\\ K_{i}=E_{i}^{\prime }W_{K}\\ V_{i}=E_{i}^{\prime }W_{V} \end{array}\right.\;$$

Among them, $Q_{i}$, $K_{i}$, and $V_{i}$ all belong to $R^{B\times L\times H}$, where B, L, and H denote the batch size, sequence length, and hidden dimension, respectively. $W_{Q}$, $W_{K}$, and $W_{V}$ are the learnable weight matrices obtained during training. The index $i\in \left\{1,2,\ldots ,\tilde{N}\right\}$, where $\tilde{N}$ represents the total number of patches obtained after dividing the input image.

(2) Multi-Head Attention Splitting

The vectors $Q_{i}$, $K_{i}$, and $V_{i}$ are divided into H subspaces (H=8). In the h−th attention head ($h\in \left[1,H\right]$), the query(Q), key(K), and value(V) corresponding to position i are denoted as ${Q_{i}}^{\left(h\right)}$, ${K_{i}}^{\left(h\right)}$, and ${V_{i}}^{\left(h\right)}$, respectively, as shown in Equation (5). Here, $W_{Q}^{\left(h\right)}$, $W_{K}^{\left(h\right)}$, and $W_{V}^{\left(h\right)}$ represent the subspace projection matrices for the h attention head.

(5) $$\left\{\begin{array}{c} {Q_{i}}^{\left(h\right)}=E_{i}^{\prime }W_{Q}^{\left(h\right)}\\ {K_{i}}^{\left(h\right)}=E_{i}^{\prime }W_{K}^{\left(h\right)}\\ {V_{i}}^{\left(h\right)}=E_{i}^{\prime }W_{V}^{\left(h\right)} \end{array}\right.$$

(3) Scaled Dot-Product Attention Computation

In the h−th attention head, the attention weight vector between the i−th token and the j−th token is denoted as $A_{i,j}^{\left(h\right)}$, as shown in Equation (6).

(6) $$A_{i,j}^{\left(h\right)}={softmax}_{j}\left(\frac{{Q_{i}}^{\left(h\right)}{\left({K_{j}}^{\left(h\right)}\right)}^{T}}{\sqrt{d_{k}}}\right)$$

Here, ${Q_{i}}^{\left(h\right)}$ represents the query vector at position i in the h−th attention head, while ${\left({K_{j}}^{\left(h\right)}\right)}^{T}$ denotes the transpose of the key vector at position j in the same head, where $i,j\in \left\{1,2,\ldots ,\tilde{N}\right\}$. A scaling factor of $\sqrt{d_{k}}$ is applied to normalize the dot-product results, which helps prevent gradient explosion or vanishing and ensures training stability. The softmax operation, denoted as ${Softmax}_{j}()$, is applied along the j-dimension to generate the attention distribution of the i−th token over all other tokens.

(4) Weighted Summation and Output

Multi-Head Attention (MHA) applies the attention weights $A_{i,j}^{\left(h\right)}$ to compute the weighted summation of the value vectors ${V_{j}}^{\left(h\right)}$, yielding the output $O_{i}^{\left(h\right)}$ at position i in the h−th attention head, as shown in Equation (7).

(7) $$O_{i}^{\left(h\right)}=\sum_{j=1}^{N}A_{i,j}^{\left(h\right)}{V_{j}}^{\left(h\right)}$$

The outputs from all attention heads are concatenated and subsequently projected back into the original representation space using a linear transformation matrix $W_{O}$, as presented in Equation (8). This operation not only ensures the consistency of feature dimensions but also enhances the integration of information across different attention heads, thereby improving the model’s ability to capture global contextual dependencies.

(8) $$H_{i}=\mathrm{Concat}\left(O_{i}^{\left(1\right)},\ldots ,O_{i}^{\left(h\right)},\ldots ,O_{i}^{\left(H\right)}\right)W_{O}$$

2. Residual Normalization

To stabilize training and enhance gradient propagation, a residual connection and layer normalization (LayerNorm) are applied after the multi-head attention. The residual connection directly adds the input $E_{i}^{\prime }$ to the output $H_{i}$, and LayerNorm is then applied to the result of this residual connection to obtain $x^{\prime }$, as shown in Equation (9).

(9) $$x^{\prime }=\mathrm{LayerNorm}\left(E_{i}^{\prime }+H_{i}\right)$$

3. Feedforward Residual Fusion

Relying solely on the self-attention mechanism is insufficient to meet the requirements for extracting complex lesion features. A feed-forward neural network (FFN) enhances the nonlinear mapping capability of the model, thereby facilitating the extraction of more sophisticated representations. The output of LayerNorm, $x^{\prime }$, is fed into the FFN, which consists of two fully connected layers, $W_{1}$ and $W_{2}$, along with a ReLU activation function. First, $x^{\prime }$ is projected into a higher-dimensional space through the fully connected layer $W_{1}$ to improve the model’s representational capacity. The projection result, $W_{1}x^{\prime }$, is then transformed via the ReLU activation and reduced back to the original feature space through the second fully connected layer $W_{2}$. This process is expressed in Equation (10).

(10) $$\hat{x}=\mathrm{ReLU}\left(W_{1}x^{\prime }\right)W_{2}$$

The resulting output of the FFN, $\hat{x}$, is added to the previous $x^{\prime }$ through a residual connection to obtain the final output of the Diagnostic Image–Text Residual Multi-Head Semantic Encoding (DIT-RMHSE), denoted as $x_{\mathrm{text}}$, as shown in Equation (11).

(11) $$x_{\mathrm{text}}=x^{\prime }+\hat{x}$$

### 2.2. Fine-Grained Multimodal Fusion with Local Attention

**Figure 3:**
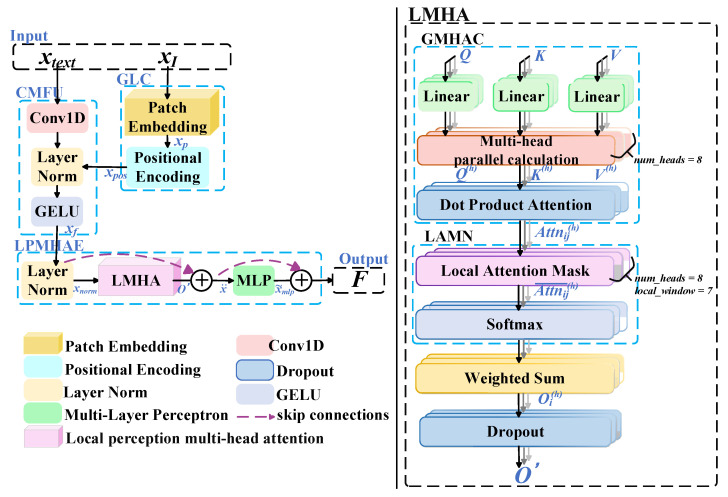
Fine-Grained Multimodal Fusion with Local Attention (FG-MFLA).

In medical image segmentation tasks, complex lesion regions often exhibit irregular boundaries and fine-grained details. Conventional neural networks demonstrate clear limitations in local feature extraction, long-range dependency modeling, and global context representation. These challenges are further amplified when processing multimodal data, where traditional approaches frequently fail to effectively integrate intricate cross-modal relationships, leading to information loss and performance bottlenecks. Such shortcomings hinder the precise segmentation of complex lesion areas in medical images. To address these issues, Fine-Grained Multimodal Fusion with Local Attention (FG-MFLA) is proposed. This approach leverages the synergy of the multi-head attention mechanism and local attention masks, in combination with the Cross-Modal Fusion Unit (CMFU), to enhance multimodal feature integration. The implementation of this module is divided into six steps, as illustrated in Figure 3.

#### 2.2.1. Generation of Positional Encoding

The medical image $x_{I}$ is partitioned into $\tilde{N}$ fixed-size patches through the patch embedding module, resulting in $x_{p,i}$, where $i\in [1,\tilde{N}]$, as shown in Equation (12).

(12) $$x_{p,i}=\mathrm{PatchEmbedding}\left(x_{I}\right)$$

Each $x_{p,i}$ is then added to a positional encoding $E_{\mathrm{pos},i}$, producing the image feature $x_{\mathrm{pos},i}$, as described in Equation (13).

(13) $$x_{\mathrm{pos},i}=x_{p,i}+E_{\mathrm{pos},i}$$

Here, $E_{\mathrm{pos},i}$ denotes a learnable positional encoding designed to retain the spatial information of each patch.

#### 2.2.2. Feature Integration and Normalization

The image features $x_{\mathrm{pos}}$, constructed from $N^{\prime }$ instances of $x_{\mathrm{pos},i}\;$($i\in [1,N^{\prime }]$), are fused with the text feature $x_{text}$ using the Cross-Modal Fusion Unit (CMFU), which consists of Conv1D, LayerNorm, and GELU. CMFU adopts a bias-free fusion strategy, allowing visual and textual features to participate in the fusion equally and complementarily. This can prevent feature competition, redundancy, or one modality dominating. This process generates the fused feature $x_{f}$, as presented in Equation (14).

(14) $$x_{f}=\mathrm{CMFU}\left(x_{\mathrm{pos}},x_{text}\right)$$

#### 2.2.3. Local Perception Multi-Head Attention Encoder

1. Feature normalization

To ensure a uniform feature distribution and reduce instability caused by gradient variations, the feature representation $x_{f}$ undergoes normalization through LayerNorm, resulting in $x_{\mathrm{norm}}$, as shown in Equation (15).

(15) $$x_{\mathrm{norm}}=\mathrm{LayerNorm}\left(x_{f}\right)$$

2. Local Perception Multi-Head Attention

Local perception multi-head attention serves as the core component of the encoder. The normalized feature $x_{\mathrm{norm}}$ is processed through the same linear transformations as in the MHA procedure of DIT-RMHSE. The multi-head parallel computation is formulated in Equations (4) and (5).

(1) Local perception multi-head attention

Following the aforementioned process, the input embedding $x_{\mathrm{norm}}$ produces attention weights in the h attention head of the H multi-head subspaces. Specifically, the attention weight of the i-th position attending to the j-th position is denoted as ${Attn}_{i,j}^{\left(h\right)}$, as shown in Equation (16).

(16) $${Attn}_{i,j}^{\left(h\right)}={Softmax}_{j}\left(\frac{Q^{\left(h\right)}\left[i\right]{K^{\left(h\right)}\left[j\right]}^{T}}{\sqrt{D_{h}}}\right)$$

Here, $Q^{\left(h\right)}\left[i\right]$ represents the query vector of the i-th position in the h-th attention head, and $K^{\left(h\right)}\left[j\right]$ denotes the key vector of the j-th position in the same head. The indices $i,j\in \left\{1,2,\ldots ,N^{\prime }\right\}$, where $N^{\prime }$ is the number of patches obtained by partitioning the input image. The Softmax operation, denoted as ${Softmax}_{j}()$, is applied along the j-dimension to generate the attention distribution of the i-th position over all other positions. The scaling factor $\sqrt{D_{h}}$ is introduced to mitigate numerical instability arising from the increase in dimensionality.

(2) Local Attention Mask Normalization

The local attention mask $M_{i,j}$ is designed to restrict the receptive field of attention computation and reduce computational complexity. For each query position i, a local window W is defined, limiting its focus to the nearby keys; here, i and j denote the query and key positions in the input sequence, respectively. This process is formulated in Equation (17).

(17) $$M_{i,j}=\left\{\begin{array}{c} 1,\;if\;\left|i-j\right|\leq \frac{W}{2}\\ 0,\;otherwise \end{array}\right.$$

Subsequently, the local attention mask $M_{i,j}$ is applied to the attention weight ${Attn}_{i,j}^{\left(h\right)}$ to emphasize information from local regions while suppressing positions outside the local scope, as shown in Equation (18). The multiplication of ${Attn}_{i,j}^{\left(h\right)}$ with the mask forces the attention scores outside the local window to zero. Thereafter, the Softmax operation is performed along the j-dimension on the h-th attention vector to normalize the weights, ensuring that the sum of attention weights equals one. The resulting normalized attention weight is denoted as $\bar{{Attn}_{𝚤,𝚥}^{\left(h\right)}}$.

(18) $$\bar{{Attn}_{𝚤,𝚥}^{\left(h\right)}}={softmax}_{j}\left({Attn}_{i,j}^{\left(h\right)}M_{i,j}\right)$$

(3) Weighted Summation

The normalized attention weight $\bar{{Attn}_{𝚤,𝚥}^{\left(h\right)}}$, obtained through the Softmax operation, is multiplied with the value vectors $V^{\left(h\right)}\left[j\right]$ at all positions from the value matrix $V^{\left(h\right)}$, which is generated by parallel multi-head computation. This weighted summation produces the output vector ${O_{i}}^{\left(h\right)}$ of the h-th attention head at query position i, as formulated in Equation (19). The output vector represents the degree to which query position i attends to key position j. This process allows each query position to adaptively aggregate contextually relevant information, thereby enhancing the representational capacity of the features.

(19) $${O_{i}}^{\left(h\right)}=\sum_{j=1}^{N}\bar{{Attn}_{𝚤,𝚥}^{\left(h\right)}}V^{\left(h\right)}\left[j\right]$$

(4) Regularization

The outputs from all attention heads are concatenated along the feature dimension and then projected back to the original feature space through a linear projection matrix $W_{o}$. A Dropout operation is subsequently applied, resulting in the fused output feature $O^{\prime }$, as defined in Equation (20).

(20) $$O^{\prime }=\mathrm{Dropout}\left(W_{o}\cdot Concat(O^{\left(1\right)},\ldots ,O^{\left(h\right)},\ldots ,O^{\left(H\right)}\right)$$

Here, $O^{\left(h\right)}$ denotes the output of the h-th attention head, where $h\in \left[1,H\right]$ with H=8. $Concat\left(\cdot \right)$ indicates concatenation along the feature dimension.

3. Information-Preserving Fusion

The normalized input $x_{\mathrm{norm}}$ from the Local Perception Multi-Head Attention Encoder is connected with the output $O^{\prime }$ of the Local Perception Multi-Head Attention (LMHA) through a residual connection to form the fused representation x¨\ddot{x}x¨, as shown in Equation (21). This operation preserves the original input information while introducing context enriched by local perception and multi-head attention, thereby enhancing both stability and representational capacity.

(21) $$\ddot{x}=x_{\mathrm{norm}}+O^{\prime }$$

4. Nonlinear Feature Transformation

The fused feature representation $\ddot{x}$ is transformed through a Multi-Layer Perceptron (MLP). The first linear transformation is performed using a fully connected layer followed by a GELU activation function, as defined in Equation (22), where $W_{1}$ denotes the weight matrix and $b_{1}$ represents the bias vector. The second linear transformation incorporates Dropout, as expressed in Equation (23), where $W_{2}$ denotes the weight matrix and $b_{2}$ represents the bias vector. To mitigate overfitting, the model randomly sets a fraction of the neuron outputs to zero during training. The resulting feature representation after Dropout, denoted as ${\tilde{x}}_{\mathrm{mlp}}$, constitutes the output of the MLP.

(22) $$x_{\mathrm{mlp}}=\mathrm{GELU}\left(W_{1}\ddot{x}+b_{1}\right)$$

(23) $${\tilde{x}}_{\mathrm{mlp}}=\mathrm{Dropout}\left(W_{2}x_{\mathrm{mlp}}+b_{2}\right)$$

5. Context-Preserving Enhancement

The output of the MLP, ${\tilde{x}}_{\mathrm{mlp}}$, undergoes a second residual connection with the intermediate feature $\ddot{x}$. The resulting representation F is defined as the final output of the Fine-Grained Multimodal Fusion with Local Attention (FG-MFLA), as shown in Equation (24). This operation prevents excessive information loss and ensures that sufficient contextual information is retained, even after multiple transformation stages.

(24) $$F=\ddot{x}+{\tilde{x}}_{\mathrm{mlp}}$$

### 2.3. Adaptive Global Compression and Focusing

**Figure 4:**
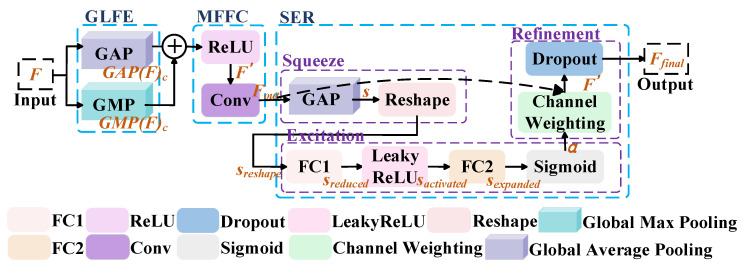
Adaptive Global Compression and Focusing (AGCF).

Existing image feature extraction methods still exhibit limitations in fine-grained feature enhancement and global information modeling, particularly when handling high-dimensional feature representations after multimodal fusion. These methods often struggle to accurately capture critical local regions. To address this issue, this thesis introduces the Adaptive Global Compression and Focusing module (AGCF), which primarily consists of three components: Multi-Scale Feature Fusion and Compression (MFFC), Global and Local Feature Extraction (GLFE), and Squeeze-and-Excitation Refinement (SER), as illustrated in Figure 4.

#### 2.3.1. Global and Local Feature Extraction

The objective of Global and Local Feature Extraction (GLFE) is to capture both global and local statistical information from the fused feature maps, thereby providing comprehensive feature sources for the subsequent fusion modules. It primarily includes two components: global statistical feature extraction and local salient feature extraction.

1. Global Statistical Feature Extraction

When processing fused multimodal features, the semantic distribution across channels is often imbalanced, which negatively impacts the model’s ability to capture overall semantics. To mitigate this problem, Global Average Pooling (GAP) is applied to compute the mean value of all spatial positions within each channel of the input feature map $F\in R^{C\times H\times W}$. This operation generates the global statistical feature $M_{1}$, as defined in Equation (25).

(25) $$M_{1}=GAP\left(F\right)$$

2. Local Salient Feature Extraction

In multimodal feature maps $F\in R^{C\times H\times W}$, salient semantic features may be unevenly distributed across different regions. To capture these variations, Global Max Pooling (GMP) is employed to compute the maximum response within each channel over the spatial dimensions (H×W). This process yields the local salient feature $M_{2}$, as defined in Equation (26).

(26) $$M_{2}=GMP\left(F\right)$$

#### 2.3.2. Multi-Scale Feature Fusion and Compression (MFFC)

During the multi-scale representation of multimodal features, issues such as feature redundancy and inconsistent semantic expressions may arise, which can degrade the quality of the fused representation. To address this problem, a Multi-Scale Feature Fusion and Compression (MFFC) method is introduced. This module operates through two main steps: global-local feature fusion and redundancy compression.

1. Global–Local Feature Fusion

Since the global statistical feature $M_{1}$ and the local salient feature $M_{2}$ differ in terms of semantic perception range and response patterns, directly fusing them may lead to redundancy or attenuation of critical features. To optimize this process, $M_{1}$ and $M_{2}$ are first fused via channel-wise addition. The fused result is then broadcast to match the spatial dimensions of the original feature map $F\in R^{C\times H\times W}$ and multiplied element-wise with F. To enhance the sparsity and discriminative capacity of feature responses, the nonlinear activation function ReLU is applied to the multiplication result, producing the refined feature map $F^{\prime }$, as defined in Equation (27).

(27) $$F^{\prime }=\mathrm{ReLU}\left(M_{1}+M_{2}\right)⊗F$$

Here, ⊗ denotes channel-wise broadcast multiplication, i.e., extending scalar values along the channel dimension to the spatial dimensions before performing element-wise multiplication with the original feature map.

2. Redundancy Compression

Although the fused feature map $F^{\prime }\in R^{C\times H\times W}$ integrates both global and local feature information, it may still contain redundant or ineffective information, which increases the parameter burden. To mitigate this issue, ${\mathrm{Conv}}_{1\times 1}$ is applied along the channel dimension to compress and refine $F^{\prime }$, thereby reducing redundancy and enhancing feature compactness, as expressed in Equation (28).

(28) $$F_{\mathrm{out}}={\mathrm{Conv}}_{1\times 1}\left(F^{\prime }\right)$$

#### 2.3.3. Channel Squeeze and Excitation Refinement (SER)

In multi-channel feature maps, different channels contribute unequally to the task. If these differences are not distinguished, it may lead to information redundancy and insufficient feature representation. To address this issue, the Channel Squeeze and Excitation Refinement (SER) method is designed to assign an adaptive weight to each channel in order to adjust its feature response, thereby enhancing the model’s focus on critical information and strengthening its representational capacity. This method consists of three main steps: feature compression, channel excitation, and feature refinement.

1. Feature Compression

The scale and information density of different input feature maps may vary, which limits the extraction of global information. To overcome this problem, a feature compression strategy is employed, in which the feature map is processed using Global Average Pooling (GAP) and shape adjustment.

(1) lobal Average Pooling

Since the spatial dimension of the feature map $F_{\mathrm{out}}$ is typically large, directly processing it may introduce redundant information and increase computational cost, thereby reducing efficiency and performance. To address this issue, GAP is applied to $F_{\mathrm{out}}$ to generate a feature map s, as shown in Equation (29).

(29) $$s=GAP\left(F_{\mathrm{out}}\right)$$

(2) Reshape

The feature map s obtained through GAP is typically in the form $\left[B,C,1,1\right]$. While this output effectively extracts global features, its spatial dimension of 1×1 does not meet the input requirements of subsequent fully connected layers. To adapt to the dimensional requirements of downstream modules, a reshape operation converts the feature map from $\left[B,C,H,W\right]$ to $\left[B,C\right]$, resulting in $s_{reshape}\in R^{B\times C}$, as shown in Equation (30).

(30) $$s_{reshape}=\mathrm{Reshape}\left(s\right)$$

2. Channel Excitation

After feature compression, the response intensity across channels may vary significantly, which can reduce the network’s ability to focus on important features. To address this issue, $s_{reshape}$ is processed through two fully connected (FC) layers to generate the final channel attention weights. The detailed steps are as follows:

(1) Dimensionality Reduction

The input feature $s_{reshape}$ is passed through the first FC layer to obtain the reduced feature $s_{reduced}\in \left[B,\frac{C}{r}\right]$. This operation decreases the number of channels, thereby removing redundant features and reducing computational cost, as shown in Equation (31).

(31) $$s_{reduced}={\mathrm{FC}}_{1}\left(s_{reshape}\right)$$

(2) Nonlinear Adaptive Activation

To enhance the expressive power of the features and to alleviate the potential limitations of linear transformations, the LeakyReLU activation function is applied to the reduced features $s_{reduced}$, producing the adaptively transformed feature $s_{activated}$, as shown in Equation (32).

(32) $$s_{activated}=\mathrm{LeakyReLU}\left(s_{reduced}\right)$$

(3) Dimensionality Restoration

The feature $s_{activated}\in \left[B,\frac{C}{r}\right]$ is then passed through the second FC layer to map it back to the original channel dimension, yielding $s_{expanded}\in R^{B\times C}$. This output represents the attention weight assigned to each channel, as shown in Equation (33).

(33) $$s_{expanded}={\mathrm{FC}}_{2}\left(s_{activated}\right)$$

(4) Normalization

The expanded output $s_{expanded}$ is normalized using a Sigmoid function, which compresses each channel’s value into the range $\left(0,1\right)$, thereby generating the channel attention weights a, as shown in Equation (34). A channel with higher importance will have an attention weight closer to 1, thereby strengthening its feature representation. Conversely, if the attention weight approaches 0, it indicates a weaker contribution, and the model reduces its influence accordingly.

(34) $$a=\mathrm{Sigmoid}\left(s_{expanded}\right)$$

3. Feature Refinement

(1) Channel Weighting

To enhance the representation of important channels while suppressing redundant or irrelevant ones, the output feature map $F_{\mathrm{out}}$ generated in Equation (28) is element-wise multiplied by the channel attention weights a. This operation produces the channel-weighted feature map $F^{\prime }$, as shown in Equation (35).

(35) $$F^{\prime }=F_{\mathrm{out}}\cdot a$$

(2) Dropout

During training, the model may risk overfitting to specific channel features. To mitigate this issue, Dropout is applied to prevent excessive reliance on certain channels. Specifically, a proportion of the channel features in $F^{\prime }$ are randomly set to zero at a predefined dropout rate (${\mathrm{dropout}}_{rate}$), thereby reducing overfitting, as shown in Equation (36).

(36) $$F_{\mathrm{drop}}=\mathrm{Dropout}\left(F^{\prime }\right)$$

However, applying Dropout during training introduces random feature removal, which decreases the overall output magnitude and may compromise both training stability and inference consistency. To address this, a scaling operation is applied: the Dropout output $F_{\mathrm{drop}}$ is multiplied by a factor of $\frac{1}{1-dropout\_rate}$. This yields the refined feature map $F_{\mathrm{final}}$, as shown in Equation (37). This scaling ensures that the expected output remains consistent with that of the non-Dropout case, thereby maintaining stability across training and inference phases.

(37) $$F_{\mathrm{final}}=\frac{F_{\mathrm{drop}}}{1-dropout\_rate}$$

## 3. Experimental Results and Analysis

### 3.1. Datasets

This thesis employs two publicly available medical image datasets, MosMedData+ [43] and QaTa-COV19 [44], to comprehensively evaluate the performance of the proposed VT-MFLV in multimodal medical image segmentation tasks.

The MosMedData+ dataset, released by medical institutions in Moscow, Russia, consists of 2729 chest CT scans from patients with COVID-19 or viral pneumonia, with 2183 images allocated for training, 273 for validation, and 273 for testing. The QaTa-COV19 dataset, jointly developed by Qatar University and research teams from Turkey, contains 9258 chest X-ray images representing healthy subjects as well as cases of pneumonia and COVID-19, including 5716 training samples, 1429 validation samples, and 2113 test samples. Both of these datasets provide pixel-level annotations of the lesion area and are both split by image for training, testing and validation.

Both the MosMedData+ and QaTa-COV19 datasets are accompanied by corresponding medical text annotation files, which cover all images in the training set, validation set, and test set. Among them, the MosMedData+ dataset contains 2729 text annotations, while the QaTa-COV19 dataset contains 9258 text annotations. Each piece of text in the text dataset corresponds one-to-one with each image in the image dataset. All text annotations were provided and verified by two experts from the Department of Radiation Oncology at the University of Texas Southwestern Medical Center [41], describing the location, quantity, and extent of the lung lesions observed in the images. In addition, a radiologist independently annotates the same group of images and compares the results to ensure the consistency and reliability of the annotations. Each Text comment file is stored in.xlsx format and includes two fields: “Image” (image file name) and “Text” (corresponding natural language description). For instance, a typical description would be: “Unilateral pulmonary infection, one infected area, lower left lung.”

### 3.2. Evaluation Metrics

For performance evaluation in medical image segmentation tasks, the Dice Similarity Coefficient (DSC) and the mean Intersection over Union (mIoU) are employed as evaluation metrics to assess the proposed VT-MFLV in terms of segmentation accuracy and regional consistency.

The Dice coefficient measures segmentation accuracy by computing the degree of overlap between the predicted infection region P and the ground truth infection region  G, as shown in Equation (38).

(38) $$Dice=\frac{2|P\cap G|}{|P|+|G|}$$

Here, P represents the infection region predicted by the model, G denotes the corresponding ground truth region in the image, and ∣P∩G∣ indicates the number of pixels in the intersection of P and G. A Dice value closer to 1 indicates higher consistency between the predicted and ground truth regions, reflecting better segmentation performance.

In addition, to further evaluate the spatial correspondence between the predicted and ground truth regions, the mean Intersection over Union (mIoU) is introduced as a complementary evaluation metric. The mIoU quantifies regional consistency by calculating the ratio between the number of pixels in the intersection P∩G and the union P∪G, as shown in Equation (39).

(39) $$mIoU=\frac{|P\cap G|}{|P\cup G|}$$

A higher mIoU value indicates a greater degree of overlap between the predicted and ground truth regions, making it a critical metric for assessing segmentation accuracy.

### 3.3. Experimental Details

Environment: This study was conducted on a workstation running the Ubuntu 20.04 operating system, with PyCharm Community 2023.2 serving as the development environment. The hardware configuration consisted of the following: (1) Intel (R) Xeon (R) W-2245 @ 3.90 GHz (CPU); (2) NVIDIA GeForce RTX 3090 (GPU), NVIDIA-SMI 470.256.02; and (3) random access memory (RAM) of 64 GB. During the experiments, PyTorch 1.8.0 and torchvision 0.9.0 were employed as the primary deep learning frameworks.

Training Parameter Configuration: The proposed VT-MFLV was systematically trained and optimized with the following parameter settings. The batch size was set to 4, and the learning rate was dynamically adjusted using a cosine annealing strategy. The initial learning rate was set to 1 × 10^−4^, with the adjustment period T_0 set to 20, the period multiplier T_mult set to 1, and the minimum learning rate lr_min set to 1 × 10^−6^. The AdamW optimizer was employed with a weight decay of 1 × 10^−4^ to ensure stable convergence during training. To enhance feature representation, a Local-aware Multi-Head Attention (LMHA) mechanism was incorporated into the visual backbone. Local attention encoded image patches using a sliding window of size 7 × 7, effectively capturing lesion boundary details, while global attention modeled long-range dependencies across regions to improve spatial semantic perception. To strengthen key channel responses, a Squeeze-and-Excitation Refinement (SER) method was introduced into the visual pathway, with the reduction ratio set to 8, thereby emphasizing lesion-related features. The Leaky ReLU activation function with a negative slope of 0.2 was employed to mitigate performance bottlenecks caused by gradient sparsity during training. We trained the model using random seeds 42, 2023 and 777, respectively, with each training session set at 200 epochs. The total parameter quantity of the model is 28.3 M, and it takes 20 h to train on NVIDIA GeForce RTX 3090 (GPU).

### 3.4. Performance Comparison and Analysis

#### 3.4.1. Comparison of Segmentation Visualization Results

1 Visualization Results on the MosMedData+ Dataset

**Figure 5:**
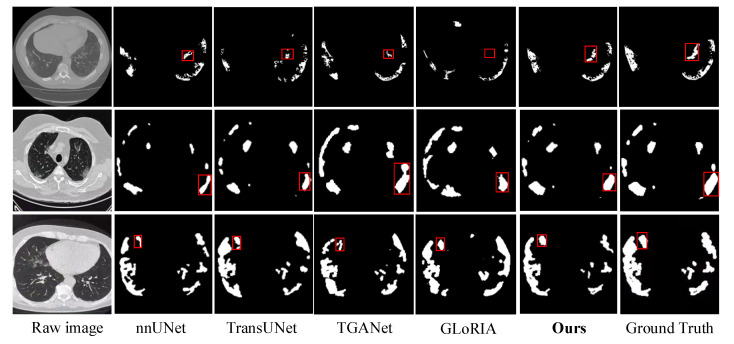
Comparison of segmentation visualization results on the MosMedData+ dataset. (The red rectangle highlights the region where the differences in segmentation performance are most visually apparent).

To further validate the fine-grained perception, structural restoration, and boundary modeling capabilities of the proposed VT-MFLV in pulmonary infection segmentation, three representative cases were selected from the MosMedData+ dataset for visualization comparison. The compared methods included nnUNet [45], TransUNet [46], TGANet [47], and GLORIA [48]. As shown in Figure 5, lesions in the MosMedData+ dataset are mostly unilateral, with clear boundaries and concentrated distributions. However, the images originate from diverse sources and are often accompanied by high noise and low contrast, which place higher demands on boundary preservation and robustness against interference.

In the first-row sample of Figure 5, the lesions exhibit small-scale, scattered patch-like structures, posing challenges for the accurate detection and localization of small targets. nnUNet segments the main lesion reasonably well but misses the smallest lesions. TransUNet and TGANet produce blurred predictions, with some small targets failing to respond. GLORIA results in missed detections in certain regions, reflecting the limitations of multimodal fusion in capturing tiny lesions. In contrast, the proposed VT-MFLV accurately identifies all lesion regions, particularly the small targets highlighted by the red boxes, demonstrating strong fine-grained structural perception. This advantage stems from the synergy between local attention and multimodal features, which effectively enhances the response strength of small lesion regions.

In the second-row sample of Figure 5, the lesions are diffusely distributed with blurred boundaries, which increases the likelihood of misjudgment in scope delineation and boundary localization. nnUNet and TransUNet both exhibit discontinuities in lesion contours. TGANet introduces significant false-positive artifacts in the central lung region. GLORIA covers most of the lesions but produces fragmented shapes. In contrast, the proposed VT-MFLV maintains structural integrity and smooth continuous boundaries, avoiding perforations and artifacts. This demonstrates the strong regional consistency achieved through image–text fusion, wherein the Diagnostic Image–Text Residual Multi-Head Semantic Encoding (DIT-RMHSE) provides crucial semantic priors.

In the third-row sample of Figure 5, lesions exhibit a multi-region distribution with complex boundary morphology and adhesion among lesions, posing challenges for boundary discrimination and multi-target separation. nnUNet and GLORIA produce fused lesions without effective boundary separation. TGANet identifies most regions but displays jagged boundaries. TransUNet generates fragmented or blurred connections. The proposed VT-MFLV achieves clear separation between lesions, preserving structural independence and closely approximating the ground-truth anatomical structures. The improved performance can be attributed to the Adaptive Global Feature Compression and Focusing (AGCF) module, which enhances boundary response regions and improves complex boundary modeling.

In summary, VT-MFLV outperforms existing methods in small-target recognition, structural preservation, and boundary restoration, demonstrating a strong semantic understanding and fine-grained structural modeling.

2 Visualization Results on the QaTa-COV19 Dataset

**Figure 6:**
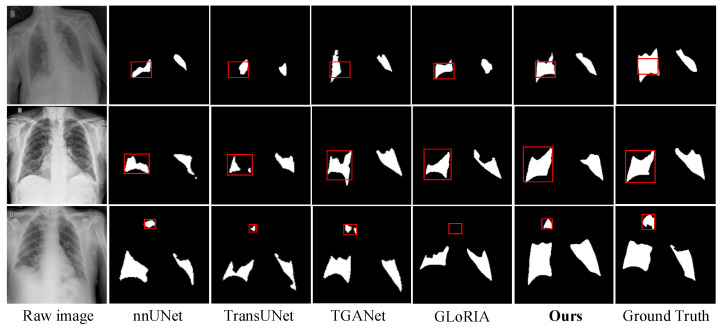
Comparison of segmentation visualization results on the QaTa-COV19 dataset. (The red rectangle highlights the region where the differences in segmentation performance are most visually apparent).

To evaluate the proposed VT-MFLV on diverse and complex lesion regions, three groups of chest X-ray images from the QaTa-COV19 dataset were used for visualization comparison with nnUNet [44], TransUNet [45], TGANet [46], and GLORIA [47]. Unlike MosMedData+, where lesions are mostly unilateral and well-defined, QaTa-COV19 lesions exhibit multi-region and multi-scale distributions, while the accompanying textual descriptions are brief, often indicating only the number and approximate location of lesions. Therefore, models must balance precise semantic perception with multi-target structural recovery. Figure 6 illustrates that VT-MFLV consistently achieves superior structural restoration and regional consistency compared with other methods.

In the first-row sample of Figure 6, lesions appear as small, isolated regions dispersed across the lungs, which may be overlooked during feature extraction. nnUNet identifies the major lesions but misses some small targets on the left. TransUNet blurs the boundaries of certain small lesions, causing fusion or fragmentation. TGANet produces regional shifts, misaligning predictions with true lesion positions. GLORIA completely fails to detect some small lesions. In contrast, the proposed VT-MFLV successfully localizes and reconstructs all critical lesions, maintaining sharp edges and accurate positions, particularly for the tiny lesion marked by the red box. This performance benefits from DIT-RMHSE, which models semantic information in textual descriptions such as “quantity” and “location,” thereby guiding the recognition of small targets.

In the second-row sample of Figure 6, lesions exhibit irregular, elongated structures with adjacent regions, necessitating strong structural consistency and boundary discrimination. nnUNet and TransUNet both display discontinuities in the central regions. TGANet inaccurately predicts the lower-left lung structure as a lesion, introducing false-positive artifacts. GLORIA incompletely segments the extended lesion in the right lung, lacking closed boundaries. In contrast, the proposed VT-MFLV reconstructs all lesions completely, avoiding breaks, adhesions, and redundant predictions, with results closely matching the ground truth. This improvement is primarily attributed to the Cross-Modal Fusion Unit (CMFU), which integrates boundary-guided mechanisms during image–text fusion, thereby enhancing structural modeling and region separation.

In the third-row sample of Figure 6, multiple lesions with significant scale differences and blurred boundaries further challenge scale adaptability and boundary delineation. nnUNet identifies large lesions but weakly responds to small regions. TransUNet produces jagged boundaries, compromising completeness. TGANet and GLORIA roughly outline major lesions but miss many small ones, leading to information loss. The proposed VT-MFLV preserves the integrity of large lesions while accurately restoring small lesions, especially the tiny lesion highlighted in the red box, which is sharply delineated. This advantage derives from the Fine-Grained Multimodal Local Attention Encoding (FG-MFLA), which strengthens the joint modeling of lesions across scales under the guidance of the attention window mechanism, enabling the unified recognition of both small and large lesions.

Overall, VT-MFLV consistently outperforms mainstream methods in small-target recognition, structural integrity, and boundary contour modeling. When applied to the QaTa-COV19 dataset, which features typical multi-region, multi-scale lesion distributions, the model still achieves high precision and highly consistent segmentation, reflecting strong multimodal synergistic modeling and robust lesion restoration capability.

#### 3.4.2. Quantitative Results Comparison

To objectively evaluate the adaptability of the proposed multimodal medical image segmentation method across different scenarios, experiments were conducted on two publicly available pulmonary medical image datasets—MosMedData+ and QaTa-COV19. Ten state-of-the-art methods were selected for quantitative performance comparison, covering unimodal approaches without textual information, including U-Net [49], AttUNet [50], nnUNet [44], TransUNet [45], Swin-UNet [51], UCTransNet [52], as well as multimodal approaches integrating textual information, including TGANet [46], CLIP [53], GLORIA [47], and VT-MFLV (Ours). The comparison results are presented in Table 1.

On the MosMedData+ dataset, lesion regions are typically concentrated, primarily manifested as medium-scale pulmonary infection areas with relatively clear boundaries, which places higher demands on the model’s ability to extract image features. This dataset is suitable for assessing a model’s capability in boundary delineation and intra-region consistency modeling. Among unimodal methods without textual information, nnUNet achieved the best results with a Dice score of 72.59% and a mean Intersection over Union (mIoU) of 60.36%. TransUNet also performed well, with a Dice score of 71.24% and an mIoU of 58.44%. In contrast, U-Net, AttUNet, and Swin-UNet achieved Dice scores of 64.58%, 66.34%, and 63.29%, and mIoU scores of 50.41%, 52.82%, and 50.19%, respectively, showing limited overall performance. UCTransNet reached a Dice score of 65.90% and an mIoU of 52.69%, slightly outperforming the aforementioned architectures but still with noticeable gaps. For multimodal methods incorporating textual information, GLORIA achieved a Dice score of 72.42% and an mIoU of 60.18%, representing the best performance among existing multimodal methods. TGANet and CLIP achieved Dice scores of 71.81% and 71.97%, and mIoU scores of 59.28% and 59.64%, respectively, performing slightly below LViT and GLORIA in structural recovery and regional consistency. In comparison, VT-MFLV achieved Dice and mIoU scores of 75.61% and 63.98%, surpassing LViT by 1.04% and 2.65%, respectively, on these two key metrics. This improvement can be attributed to the synergistic effects of multiple modules. The integration of the Fine-Grained Multimodal Fusion Local Attention (FG-MFLA) within the visual backbone enabled deep fusion of local and global image features, enhancing boundary representation and effectively capturing structural information of medium-scale lesions. During text processing, the Diagnostic Image–Text Residual Multi-Head Semantic Encoding (DIT-RMHSE) extracted semantic features that provided structural and positional priors during fusion, compensating for ambiguous regions in image-only features. Additionally, the Squeeze-and-Excitation Refinement (SER) within the Adaptive Global Compression Focusing (AGCF) mechanism adaptively reinforced boundary responses across different levels, further improving the accuracy of regional reconstruction. Consequently, in the MosMedData+ dataset, characterized by relatively regular lesions and sufficient image information, the proposed method achieved superior performance on both metrics, demonstrating robust structural awareness and segmentation accuracy.

On the QaTa-COV19 dataset, lesion morphology is markedly more complex, often presenting as small patches, scattered distributions, and blurred boundaries, which increase the challenges for segmentation models in multi-region modeling and semantic understanding. Among unimodal methods, nnUNet remained the most stable, with Dice and mIoU scores of 80.42% and 70.81%, respectively. TransUNet followed closely with scores of 78.63% and 69.13%. U-Net and AttUNet performed similarly, with Dice scores of 79.02% and 79.31% and mIoU scores of 68.76% and 70.04%. Swin-UNet and UCTransNet achieved Dice scores of 78.07% and 79.15% and mIoU scores of 67.31% and 69.60%, respectively, indicating relatively weaker performance and highlighting the limitations of unimodal visual methods in handling complex morphologies. With the integration of textual information, multimodal methods demonstrated stronger semantic alignment. With the integration of textual information, LViT achieved Dice and mIoU scores of 83.66% and 75.11%, while TGANet achieved 79.87% and 70.75%, CLIP achieved 79.81% and 69.66%, and GLORIA reached 79.94% and 70.68%. These multimodal methods demonstrated stronger semantic alignment than unimodal architectures. In comparison, VT-MFLV achieved the best results with a Dice score of 83.34% and an mIoU of 72.09%, slightly below LViT in Dice but surpassing all other methods, reflecting the effectiveness of the finely designed multimodal fusion mechanism. The contextual text features extracted by DIT-RMHSE accurately aligned multi-region semantic information, providing guidance for lesion location and category while compensating for deficiencies in visual pathways when recognizing scattered patches. During feature fusion, FG-MFLA employed multi-scale attention mechanisms to enhance fine-grained structural perception, strengthening structural modeling under complex morphologies. When processing small lesions and blurred boundaries, SER further compressed and emphasized critical response regions after multimodal fusion, significantly enhancing robustness. The joint contributions of these modules allowed the proposed method to maintain high accuracy and consistency even in the presence of multi-region, weak-boundary, and variable lesion patterns.

In summary, the proposed VT-MFLV demonstrated superior performance on both the MosMedData+ and QaTa-COV19 datasets. Particularly on the QaTa-COV19 dataset, characterized by complex structures and ambiguous semantics, it achieved notable advantages, fully showcasing the contributions of its modules in improving segmentation accuracy and generalization capability under diverse scenarios.

#### 3.4.3. Statistical Significance Evaluation

To ensure that the observed performance improvement was not due to random initialization or dataset differences, we independently trained VT-MFLV three times using different random seeds (42, 2023, and 777), along with the classical model U-Net and the current state-of-the-art model LViT. The mean ± standard deviation of Dice and mIoU were calculated, and paired two-sample *t*-tests were used to assess statistical significance. As shown in Table 2, VT-MFLV consistently outperformed U-Net on both the MosMedData and QaTa-COV19 datasets, while performing close to the state-of-the-art method LViT. On the MosMedData dataset, VT-MFLV achieved Dice and mIoU scores of 75.61 ± 0.32% and 63.98 ± 0.29%, demonstrating an improvement over LViT (74.57 ± 0.39% and 61.33 ± 0.33%) and an increase compared to U-Net (64.58 ± 0.37% and 50.41 ± 0.31%). On the QaTa-COV19 dataset, VT-MFLV achieved Dice and mIoU scores of 83.34 ± 0.36% and 72.09 ± 0.30%, slightly below LViT (83.66 ± 0.38% and 75.11 ± 0.39%), yet still showing a clear enhancement over U-Net (78.45 ± 0.40% and 68.76 ± 0.33%).

These results indicate that VT-MFLV achieves stable and statistically reliable segmentation performance under different random initializations and is already at the forefront of the field.

#### 3.4.4. Computational Efficiency Evaluation

To evaluate the computational efficiency, we conducted a comparative analysis of whether the model uses text information, parameter scale, and inference time, as shown in Table 3. Among them, the lower values of the Parameters and Inference Time indicators are both preferred.

The proposed VT-MFLV has a parameter of 28.3 M, which is larger than the traditional U-Net (14.8 M), but slightly lower than LViT (29.7 M). This indicates that although VT-MFLV, like LViT, introduces text encoders and multimodal fusion mechanisms, its overall structure still maintains a relatively compact scale. In terms of inference speed, VT-MFLV achieved an inference speed of 30.7 ms/image (512 × 512), which was only slightly slower than U-Net (25.5 ms/image), but significantly better than LViT (37.8 ms/image). It reflects a more reasonable performance trade-off between computational overhead and multimodal expression capabilities.

It is worth noting that both VT-MFLV and LViT utilize medical text descriptions as supplementary information. However, compared with LViT, VT-MFLV demonstrates superior resource efficiency in terms of model complexity and inference delay, effectively reducing computational costs while maintaining multimodal inference capabilities.

#### 3.4.5. Window Size Selection in Locality-Aware Multi-Head Attention

To further investigate the influence of the locality-aware attention window size on segmentation performance, we conducted experiments with window sizes of 3 × 3, 5 × 5, 7 × 7, and 9 × 9 on the MosMedData+ and QaTa-COV19 datasets. Each configuration was trained independently three times using different random seeds, and the results are reported as the mean ± standard deviation in Table 4. As shown, the Dice and mIoU scores generally improve as the window size increases from 3 × 3 to 7 × 7, indicating that moderate enlargement of the local receptive field helps the model capture more contextual dependencies without losing fine-grained detail. When the window expands to 9 × 9, performance slightly decreases, likely due to redundant background information being included within the attention scope. Overall, the 7 × 7 configuration achieves the best balance between local sensitivity and contextual awareness, validating its adoption as the default setting in VT-MFLV.

On the MosMedData+ dataset, when the window size was set to 3 × 3, the Dice and mIoU scores were 73.21 ± 0.22% and 62.76 ± 0.28%, respectively. Increasing the window to 5 × 5 led to a clear improvement, reaching 75.28 ± 0.19% and 63.84 ± 0.24%. The best performance was obtained with a 7 × 7 window, yielding a Dice of 75.61 ± 0.17% and an mIoU of 64.03 ± 0.20%, which demonstrates that a moderately expanded receptive field enables more accurate regional perception and boundary modeling. When the window was further enlarged to 9 × 9, the performance slightly declined to 75.11 ± 0.25% and 63.42 ± 0.21%, likely due to redundant background information diluting lesion-relevant attention.

A similar pattern was observed on the QaTa-COV19 dataset. With the 3 × 3 window, Dice and mIoU reached 80.53 ± 0.31% and 70.41 ± 0.27%, respectively, and improved to 82.35 ± 0.26% and 71.36 ± 0.29% at 5 × 5. The 7 × 7 configuration again achieved the best results, with Dice and mIoU of 83.29 ± 0.22% and 72.10 ± 0.25%, indicating a robust capacity to model spatial dependencies across regions. When expanded to 9 × 9, performance slightly dropped to 82.07 ± 0.30% and 71.08 ± 0.26%, suggesting that excessively large windows incorporate non-informative background cues.

Across both datasets, the 7 × 7 window provided the most consistent and accurate results, confirming that this configuration offers the optimal balance between local spatial detail preservation and global contextual understanding.

#### 3.4.6. Ablation Studies

To investigate the independent contributions and synergistic effects of the modules in VT-MFLV, we conducted a series of ablation studies by selectively removing key modules, namely Diagnostic Imaging Text Residual Multi-Head Semantic Encoding (DIT-RMHSE), Fine-Grained Multimodal Fusion with Local Attention (FG-MFLA), and Adaptive Global Compression and Focusing (AGCF). Evaluations were performed on both the MosMedData+ and QaTa-COV19 datasets, and the results are presented in Table 5.

To assess the actual role of textual information in image–text segmentation, we removed DIT-RMHSE from the complete model, retaining only the image branch for training and inference while keeping the rest of the architecture unchanged. Comparing segmentation performance with and without textual guidance verifies the effectiveness of textual descriptions in directing the model’s focus on lesion regions and enhancing semantic understanding. Results show that, upon removing DIT-RMHSE, the Dice score on MosMedData+ dropped from 75.61% to 72.73%, and the mIoU decreased from 63.98% to 61.42%. On QaTa-COV19, the Dice score and mIoU fell to 80.92% and 69.34%, respectively. These results confirm that semantic information effectively guides the model in focusing on key regions, particularly improving discrimination in complex infected areas.

To further assess the role of the fine-grained multimodal alignment mechanism, we removed the FG-MFLA module. Experimental results show that on the MosMedData+ dataset, the Dice score dropped from 75.61% to 73.24%, and mIoU decreased from 63.98% to 61.75%; on the QaTa-COV19 dataset, Dice and mIoU also dropped to 81.12% and 69.71%, respectively. These results indicate that FG-MFLA makes a crucial contribution to enhancing fine-grained cross-modal alignment. Since FG-MFLA can reduce semantic ambiguity through local attention constraints and strengthen spatial structure modeling, removing this module significantly reduces the model’s sensitivity to lesion boundaries and sparse structures. Overall, the results demonstrate that FG-MFLA is an important component for improving multimodal fusion quality and fine-grained spatial reasoning capability.

To evaluate the role of the adaptive channel recalibration mechanism within the overall framework, we removed the AGCF module. Experimental results show that on the MosMedData+ dataset, the Dice score dropped from 75.61% to 73.68%, and the mIoU decreased from 63.98% to 62.09%. On the QaTa-COV19 dataset, Dice and mIoU dropped to 81.46% and 70.05%, respectively. This performance decline indicates that AGCF plays a key role in suppressing redundant background information and enhancing channel responses related to lesions. Compared with the complete model, removing AGCF weakens channel discrimination capability and feature consistency, further demonstrating that this module is essential for improving the quality of feature representation after cross-modal fusion.

In the ablation experiments, each module contributed to improving segmentation performance. Among them, removing the DIT-RMHSE caused the largest performance drop, indicating that this module has the greatest impact on segmentation accuracy. This suggests that medical text descriptions play an important role in guiding the model to identify lesion structures, making DIT-RMHSE the most influential component in our method.

## 4. Conclusions

This thesis introduces the Vision–Text Multimodal Feature Learning V Network (VT-MFLV) for medical image segmentation. To enhance the semantic guidance of medical text on visual features, VT-MFLV incorporates the Diagnostic Imaging Text Residual Multi-Head Semantic Encoding (DIT-RMHSE) module, which effectively models contextual semantic relationships and improves lesion localization. To address the insufficient interaction of cross-modal features, the model employs the Fine-Grained Multimodal Local Attention Encoding (FG-MFLA) module. Building on the multi-head attention mechanism, this module integrates a local attention mask and a cross-modal fusion unit, enabling fine-grained feature modeling and improving cross-modal representation capability. To further increase the discriminability of critical regions, the Adaptive Global Compression and Focusing (AGCF) module is introduced. By applying channel compression excitation and pixel-level feature enhancement, AGCF adaptively adjusts channel weights, effectively suppressing redundant background information and highlighting lesion areas. VT-MFLV utilizes the complementary information of medical images and texts to achieve precise identification and fine-grained segmentation of key lesions, reaching the international leading level on both public pulmonary infection datasets. In future work, we will evaluate the segmentation performance separately based on the size of the lesion, verify the risk of model overfitting, and introduce boundary sensitive indicators such as Hausdorff Distance (HD95) to improve the performance evaluation. In addition, improving the robustness of the model remains an important direction for future research. Related works such as adaptive watermarking, hybrid spatial-frequency modeling, and robust feature coding [54,55,56] indicate that enhancing the stability of feature expression under disturbance conditions can provide beneficial insights for improving the consistency and reliability of multimodal medical image segmentation in complex environments such as noise, compression, or acquisition differences.

## Figures and Tables

**Table 1:** Quantitative Results Comparison. ”×” indicates no text information, “√” indicates text information.

Text	Method	MosMedData+		QaTa-COV19	
		Dice (%)	mIoU (%)	Dice (%)	mIoU (%)
×	U-Net [49]	64.58 ± 0.37	50.41 ± 0.31	78.45 ± 0.40	68.76 ± 0.33
×	AttUNet [50]	66.34	52.82	79.31	70.04
×	nnUNet [44]	72.59	60.36	80.42	70.81
×	TransUNet [45]	71.24	58.44	78.63	69.13
×	Swin-UNet [51]	63.29	50.19	78.07	68.34
×	UCTransNet [52]	65.90	52.69	79.15	69.60
√	TGANet [46]	71.81	59.28	79.87	70.75
√	CLIP [53]	71.97	59.64	79.81	69.66
√	GLoRIA [47]	72.42	60.18	79.94	70.68
√	LViT [41]	74.57 ± 0.39	61.33 ± 0.33	83.66 ± 0.38	75.11 ± 0.39
√	VT-MFLV (ours)	75.61 ± 0.32	63.98 ± 0.29	83.34 ± 0.36	72.09 ± 0.30

The sources of the results in the table include publicly available literature data (AttUNet, nnUNet, TransUNet, Swin-UNet, UCTransNet, TGANet, CLIP, GLoRIA) reported in Li et al. [41], TMI 2024 and the statistical data of retraining three times using the official code (U-Net, LViT).

**Table 2:** Statistical Comparison.

Dataset	Model	Dice (%)	mIoU (%)	*p*-Value (Dice)	*p*-Value (mIoU)
MosMedData+	U-Net	64.58 ± 0.37	50.41 ± 0.31	—	—
	LViT	74.57 ± 0.39	61.33 ± 0.33	—	—
	VT-MFLV	75.61 ± 0.32	63.98 ± 0.29	0.0008	0.0006
QaTa-COV19	U-Net	78.45 ± 0.40	68.76 ± 0.33	—	—
	LViT	83.66 ± 0.38	75.11 ± 0.39	—	—
	VT-MFLV	83.34 ± 0.36	72.09 ± 0.30	0.0011	0.0010

**Table 3:** Computational Efficiency Comparison.”×” indicates no text information, “√” indicates text information.

Model	Text	Parameters (M)	Inference Time (ms/Image)
U-Net	×	14.8	25.5
LViT	√	29.7	37.8
VT-MFLV (ours)	√	28.3	30.7

**Table 4:** Window size selection experiments for LMHA.

Window Size	MosMedData+		QaTa-COV19	
	Dice (%)	mIoU (%)	Dice (%)	mIoU (%)
3 × 3	73.21 ± 0.22	62.76 ± 0.28	80.53 ± 0.31	70.41 ± 0.27
5 × 5	75.28 ± 0.19	63.84 ± 0.24	82.35 ± 0.26	71.36 ± 0.29
7 × 7 (Ours)	75.61 ± 0.17	64.03 ± 0.20	83.29 ± 0.22	72.10 ± 0.25
9 × 9	75.11 ± 0.25	63.42 ± 0.21	82.07 ± 0.30	71.08 ± 0.26

**Table 5:** Ablation Studies.

Method	MosMedData+		QaTa-COV19	
	Dice (%)	mIoU (%)	Dice (%)	mIoU (%)
FG-MFLA +AGCF	72.73	61.42	80.92	69.34
DIT-RMHSE+AGCF	73.24	61.75	81.12	69.71
DIT-RMHSE+ FG-MFLA	73.68	62.09	81.46	70.05
VT-MFLV (Ours)	75.61	63.98	83.34	72.09

## Data Availability

The data presented in this study are openly available in MosMedData+ [43] and QaTa-COV19 [44] at http://medicalsegmentation.com/covid19/ (accessed on 15 October 2025), and https://www.kaggle.com/datasets/aysendegerli/qatacov19-dataset (accessed on 15 October 2025). Source code of our VT-MFLV method can be downloaded at https://github.com/JIAQILITech/VT-MFLV (accessed on 24 October 2025).

## Appendix A

### Appendix A.1

To ensure clarity and consistency throughout the manuscript, Table A1, “Notation and Symbol Definitions,” provides a unified description of all mathematical symbols and variables. The table includes token representations, embedding dimensions, attention parameters, and computational operators adopted in the proposed multimodal segmentation framework. This reference is intended to assist readers in accurately interpreting the model formulation and experimental methodology.

**Table A1:** Notation and Symbol Definitions.

Symbol	Meaning
N	Number of tokens after tokenization
L	Length of the original character sequence
d	Embedding dimension
pos	Position index
H	Number of attention heads
V	Vocabulary size
Z	Output of MHSA module
A	Attention map
⊕	Element-wise addition/
⊗	multiplication

### Appendix A.2

Table A2 provides representative examples of textual annotations used in the VT-MFLV model. These descriptions were obtained from the text-annotation files released in the LViT repository (https://github.com/HUANGLIZI/LViT (accessed on 15 October 2025)) corresponding to the MosMedData+ and QaTa-COV19 datasets. As stated in the LViT publication [41], the textual annotations were provided and verified by two professionals from the Department of Radiation Oncology, UT Southwestern Medical Center, who independently annotated the same images and compared their descriptions to ensure consistency. Each example below includes the image identifier and its corresponding clinical text description. These samples illustrate the type and quality of textual input used for multimodal learning.

**Table A2:** Example Text Descriptions Used in VT-MFLV.

Image ID	Text Description
Morozov_study_0266_24.png	Unilateral pulmonary infection, one infected area, left lung and middle right lung.
Jun_coronacases_case4_143.png	Unilateral pulmonary infection, one infected area, left lung and middle right lung.
Morozov_study_0263_2.png	Unilateral pulmonary infection, one infected area, left lung and middle right lung.
Jun_radiopaedia_14_85914_0_case13_19.png	Bilateral pulmonary infection, two infected areas, upper left lung and upper right lung.
Morozov_study_0275_12.png	Unilateral pulmonary infection, one infected area, middle left lung.
Jun_coronacases_case6_93.png	Unilateral pulmonary infection, one infected area, middle left lung.
Morozov_study_0276_22.png	Unilateral pulmonary infection, one infected area, middle left lung.
Jun_coronacases_case8_177.png	Unilateral pulmonary infection, two infected areas, upper left lung.
Jun_coronacases_case8_269.png	Unilateral pulmonary infection, one infected area, middle left lung.
Jun_coronacases_case8_226.png	Bilateral pulmonary infection, three infected areas, upper left lung and middle right lung.
Morozov_study_0296_12.png	Bilateral pulmonary infection, two infected areas, upper left lung and middle right lung.
Morozov_study_0296_13.png	Unilateral pulmonary infection, four infected areas, upper left lung.
Jun_coronacases_case8_70.png	Bilateral pulmonary infection, two infected areas, middle left lung and middle right lung.
Morozov_study_0296_11.png	Unilateral pulmonary infection, one infected area, upper left lung.
Jun_coronacases_case8_192.png	Unilateral pulmonary infection, three infected areas, upper left lung.
